# Public health round-up

**DOI:** 10.2471/BLT.14.011114

**Published:** 2014-11-01

**Inspiration from the world’s best road safety videos**

Countries interested in launching a new road safety campaign can now refer to a unique collection of 60 public information videos in nine languages from 13 countries, considered some of the best in the world. “We hope that these videos will inspire countries wanting to develop their own road safety campaigns,” said Dr Etienne Krug, director of WHO’s department of the Management of NCDs, Disability, Violence and Injury Prevention. The photo shows a still from a video from Turkey.

http://www.who.int/violence_injury_prevention/videos

**Figure:**
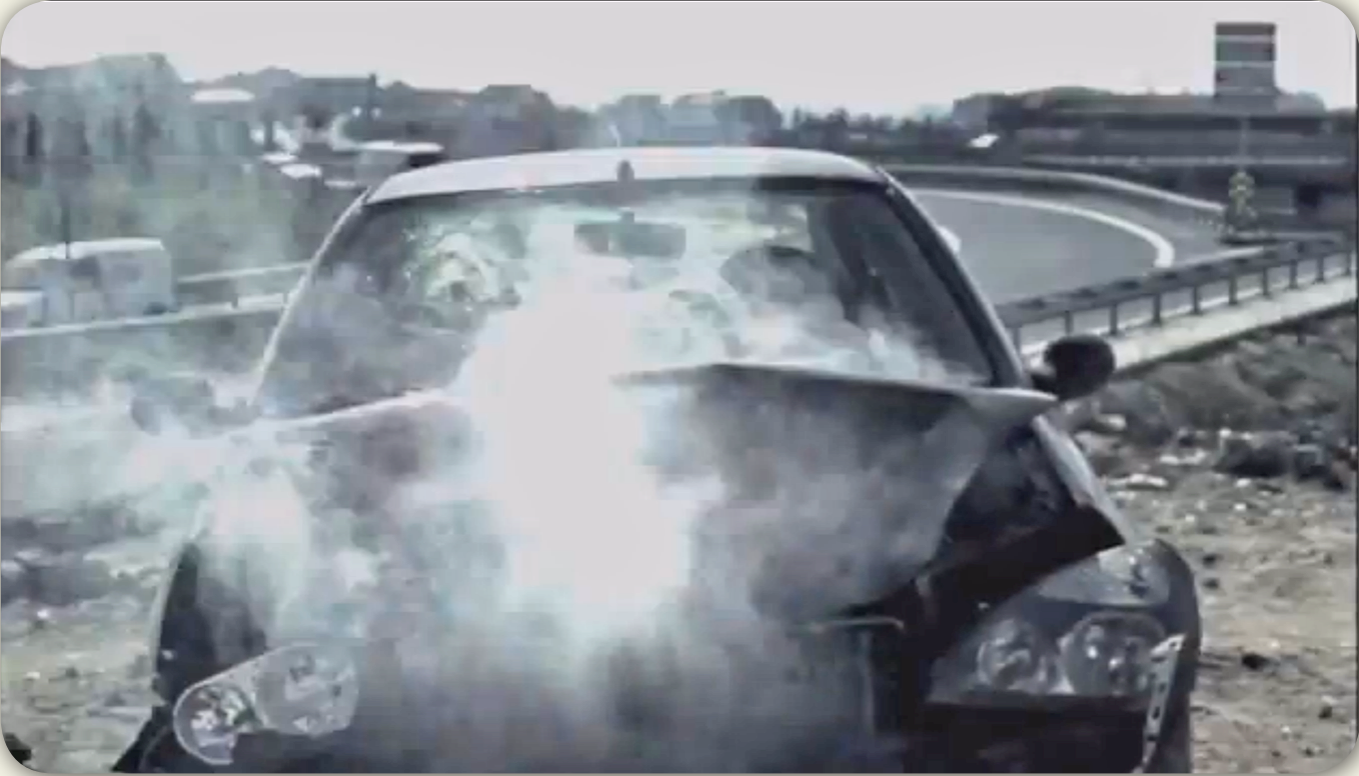
WHO

## Ebola vaccine trials start

The first clinical trials of an experimental vaccine for Ebola virus disease in Africa started last month in Mali to test its safety and ability to provoke an immune response, or immunogenicity.

News of this and other vaccine trials came as the international community ramped up efforts to stop the outbreak that is ravaging parts of western Africa.

Clinical trials to test the safety and immunogenicity of the two leading Ebola vaccine candidates (ChimpAd3-EBO and rVSV-ZEBO) were also due to start in Switzerland last month. Both were to recruit about 120 volunteers with the aim of determining the vaccine dose level needed in subsequent trials to test the vaccines’ efficacy in western Africa.

Usually clinical trials take years to complete, but if all goes well, one or more of these vaccines could be ready in several months’ time to protect front-line workers and the community in the current outbreak in western Africa.

The two vaccine candidates were reviewed by more than 70 international experts, gathered at WHO headquarters in Geneva on 29 and 30 September. The ChimpAd3-EBO vaccine is being developed by GlaxoSmithKline in collaboration with the US National Institute of Allergy and Infectious Diseases and is also being tested in the United Kingdom and the United States of America. The rVSV-ZEBO vaccine is being developed by US pharmaceutical company NewLink Genetics and the Public Health Agency of Canada. Both vaccines are currently in clinical trials in the United States.

The expert meeting also recommended that convalescent blood and plasma therapies be prioritized for further investigation. First used in small studies in previous Ebola virus disease outbreaks, but with inconclusive results, convalescent whole blood from survivors has been used to treat patients in the current outbreak in line with new WHO guidelines.

“So far, however, there is too little data to support any conclusions about the efficacy of this treatment since very few people have been treated in this way. Moreover, blood services in the affected countries are weak and lack the infrastructure to implement the WHO guidelines,” said Dr Marie-Paule Kieny, Assistant Director General at WHO for the Health Systems and Innovation cluster of departments at WHO headquarters in Geneva.

“WHO is working with international partners and each of the affected countries to develop operational plans to implement these guidelines as a matter of urgency,” Kieny said.

As of 11–12 October, 8973 cases had been reported in Guinea, Liberia and Sierra Leone, the three worst affected countries, and 4484 deaths.

http://www.who.int/csr/disease/ebola

## UN mission to Kenya

A United Nations mission to Kenya last month found high levels of obesity, smoking, physical inactivity and alcohol consumption that can lead to noncommunicable diseases (NCDs), such as heart disease, stroke, diabetes and cancer.

The Kenyan mission was the second in a series planned by the Joint Mission of the United Nations Interagency Task Force on the Prevention and Control of NCDs. The first such mission was to Belarus in July.

United Nations Country Teams are providing support for governments throughout the world in their efforts to tackle the rising epidemic of NCDs. The Interagency Task Force provides additional support for this process, working closely during its missions with the governments and the UN country teams.

The Task Force is coordinating support from United Nations and other organizations in line with the 2011 UN Political Declaration on NCDs as well as the 2014 UN Outcome Document on NCDs. 

The goal is to implement the WHO Global Action Plan for the Prevention and Control of Noncommunicable Diseases 2013–2020, which calls for a reduction in the number of premature deaths from cancer, heart disease and stroke, diabetes and respiratory diseases by 25% by 2025.

In Kenya, officials expressed concern about the high level of risk factors for these diseases. 

The estimated prevalence of smoking among men in Kenya is 26%, about 30% of Kenyan adults are overweight and 9% obese, and heavy episodic drinking of alcohol is reported to be high.

http://www.who.int/nmh/ncd-task-force

**Cover photo**

A pregnant woman is transported to hospital in Cambodia. This photo is one of a series called Birth Day, by Belgian photographer Lieve Blancquaert, telling 14 stories about childbirth from different cultures around the world. This and other photos from the collection were exhibited at WHO headquarters in Geneva this year.

**Figure:**
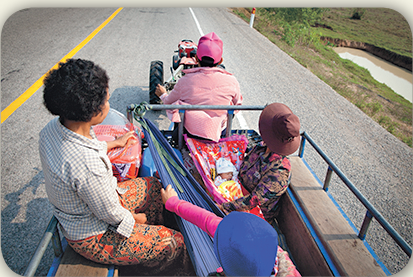
Courtesy of Lieve Blancquaert

## Age-friendly cities

Cities and communities around the world are increasingly taking action to become more age-friendly. Last month, WHO launched a new dedicated website, called Age-friendly World, to support them in these efforts.

An age-friendly city adapts its services and infrastructure so that older people with varying needs and capacities can use them, according to a recent WHO guide on the subject. The idea is to encourage people as they become older to stay active and to give them opportunities to participate in the community.

The new website provides guidance and tools that cities and communities can use when implementing and evaluating age-friendly initiatives. It also provides a forum, where they can share information on projects that are already up and running around the world.

http://agefriendlyworld.org

## Top prize for WHO book 

The second edition of WHO’s *Pocketbook for hospital care for children* has won first prize in the paediatrics category and been nominated Medical Book of the Year 2014 by the British Medical Association (BMA).

The jury called the book “an authoritative source of information about management of sick children in hospital in developing countries” and “an invaluable guide for health professionals”, concluding: “It is issued by WHO, so it’s the benchmark to be used”.

Three other WHO books were “highly commended” in the public health category: *Systematic screening for active tuberculosis*, *International perspectives on spinal cord injury* and the *Handbook on health inequality monitoring*.

http://www.who.int/maternal_child_adolescent/documents/9241546700

## Using data to stop TB

Health information systems in countries provide a rich source of data on the burden of disease caused by tuberculosis and the effectiveness of programmatic efforts to reduce this burden. But often these data are underused or not used at all by countries in their disease control efforts.

A new WHO handbook, entitled *Understanding and using TB data*, seeks to address this by giving practical examples of how to analyse tuberculosis surveillance and notification data, surveillance data for drug-resistant tuberculosis as well as tuberculosis mortality data from national vital registration systems.

http://www.who.int/tb/publications/understanding_and_using_tb_data

## Tobacco control measures

Parties to the WHO Framework Convention on Tobacco Control (FCTC) called for tighter regulation of electronic cigarettes (e-cigarettes), including banning them or restricting their promotion, advertising and sponsorship. 

The move came at the sixth session of the Conference of Parties (COP) in Moscow from 13 to 18 October in response to a WHO report released at the end of August proposing regulatory action to limit the use of e-cigarettes. These public health measures aim to stop people starting to use e-cigarettes as well as to protect bystanders from inhaling their vapours. 

The COP, the governing body of the international treaty comprising 179 countries, decided that tax rates should be monitored, increased and adjusted annually, taking inflation and income growth into account, as tobacco taxation is a known means of reducing smoking. Such measures should apply to all tobacco products including water pipes and smokeless tobacco, the COP agreed.

The 179 countries also decided that tobacco products should be taxed in a comparable way to prevent substitutions of the use of one product with another. 

Measures to restrict interference from the tobacco industry in control efforts were also decided. They include a request to the Convention Secretariat, at the WHO headquarters in Geneva, to continue providing technical support to countries that are parties to the FCTC and to engage with international organizations on the matters of tobacco companies’ influence. 

The COP made recommendations for the Parties to ratify, accede, formally confirm or approve the Protocol to Eliminate Trade in Trade Products for its entry into force, which will take place once 40 Parties have joined. The COP also proposed measures to promote sustainable alternative livelihoods for tobacco growers. 

The COP meets regularly to review progress that countries have made in implementing the Convention, and it takes decisions needed to expedite this. 

“Parties have taken courageous steps forward in a number of areas and I am pleased by the guidance to the Secretariat to scale up our collaboration with international organizations to reduce tobacco use, while continuing to assist Parties in accelerating the implementation of the Treaty,” said Dr Vera da Costa e Silva, Head of the Convention Secretariat.

**Looking ahead**

**1 December – World AIDS Day** http://www.worldaidsday.org

**3–6 December – World Cancer Congress in Melbourne, Australia** http://www.worldcancercongress.org

**26 January–3 February 2015 – WHO Executive Board meeting in Geneva, Switzerland** http://apps.who.int/gb/e/e_eb136.html

